# Regioselective One-Pot Synthesis of Hydroxy-(*S*)-Equols Using Isoflavonoid Reductases and Monooxygenases and Evaluation of the Hydroxyequol Derivatives as Selective Estrogen Receptor Modulators and Antioxidants

**DOI:** 10.3389/fbioe.2022.830712

**Published:** 2022-03-24

**Authors:** Hanbit Song, Pyung-Gang Lee, Junyeob Kim, Joonwon Kim, Sang-Hyuk Lee, Hyun Kim, Uk-Jae Lee, Jin Young Kim, Eun-Jung Kim, Byung-Gee Kim

**Affiliations:** ^1^ School of Chemical and Biological Engineering, Seoul National University, Seoul, South Korea; ^2^ Institute of Molecular Biology and Genetics, Seoul National University, Seoul, South Korea; ^3^ Institute of Engineering Research, Seoul National University, Seoul, South Korea; ^4^ Bio-MAX/N-Bio Institute, Seoul National University, Seoul, South Korea; ^5^ Institute for Sustainable Development (ISD), Seoul National University, Seoul, South Korea

**Keywords:** hydroxy-(S)-equol, oxidoreductases, isoflavonoids, SERM (selective estrogen receptor modulator), antioxidants, one-pot synthesis, enzyme engineering

## Abstract

Several regiospecific enantiomers of hydroxy-(*S*)-equol (HE) were enzymatically synthesized from daidzein and genistein using consecutive reduction (four daidzein-to-equol–converting reductases) and oxidation (4-hydroxyphenylacetate 3-monooxygenase, HpaBC). Despite the natural occurrence of several HEs, most of them had not been studied owing to the lack of their preparation methods. Herein, the one-pot synthesis pathway of 6-hydroxyequol (6HE) was developed using HpaBC (*Ec*HpaB) from *Escherichia coli* and (*S*)-equol-producing *E. coli*, previously developed by our group. Based on docking analysis of the substrate or products, a potential active site and several key residues for substrate binding were predicted to interpret the (*S*)-equol hydroxylation regioselectivity of *Ec*HpaB. Through investigating mutations on the key residues, the T292A variant was verified to display specific mono-*ortho*-hydroxylation activity at C6 without further 3′-hydroxylation. In the consecutive oxidoreductive bioconversion using T292A, 0.95 mM 6HE could be synthesized from 1 mM daidzein, while 5HE and 3′HE were also prepared from genistein and 3′-hydroxydaidzein (3′HD or 3′-ODI), respectively. In the following efficacy tests, 3′HE and 6HE showed about 30∼200-fold higher EC_50_ than (*S*)-equol in both ER_α_ and ER_β_, and they did not have significant SERM efficacy except 6HE showing 10% lower β/α ratio response than that of 17β-estradiol. In DPPH radical scavenging assay, 3′HE showed the highest antioxidative activity among the examined isoflavone derivatives: more than 40% higher than the well-known 3′HD. In conclusion, we demonstrated that HEs could be produced efficiently and regioselectively through the one-pot bioconversion platform and evaluated estrogenic and antioxidative activities of each HE regio-isomer for the first time.

## Introduction

Anaerobic equol-producing bacteria such as *Slackia isoflavoniconvertens*, *Eggerthella* sp. YY7918, and *Lactococcus garvieae* can convert daidzein (or genistein) into (*S*)-equol (or (*-*)-5-hydroxyequol) in the human intestine with a strict enantioselective manner (Uchiyama et al., 2007; Yokoyama and Suzuki, 2008; Matthies et al., 2012). When such gut bacterial metabolites reach their biological target organs or tissues, they function as a selective estrogen receptor modulator (SERM) based on their selective binding affinity for ER_β_ over ER_α_ (Setchell et al., 2005). The functional phytoestrogen (*S*)-equol has been clinically verified to be effective against estrogen or testosterone-related health problems including women’s menopausal symptoms, ovarian/prostate cancers, osteoporosis, and even hair loss (Lund et al., 2004; Jackson et al., 2011). However, its metabolism in the human body has not been completely understood yet. R.J. Schwen *et al.* observed that (*S*)-equol in rats, monkeys, and humans is mainly metabolized through the 4′-glucuronide conjugated form or 7-sulfated form in minor (Schwen et al., 2012). Otherwise, most researchers believe that it is just diluted and eventually removed along with urine, but a study suggests that hydroxylation of equol in the liver might be another route to discard equol in the human body (Marrian and Haslewood, 1932; Rufer et al., 2006). According to the observation, equol is mainly hydroxylated at C-3′, six or 8 *ortho*-positions of its inherent hydroxyl groups by enzymes, possibly cytochrome P450s (CYPs) present in the liver microsome.

Phytochemicals comprising multiple phenol groups on their backbone are called ‘polyphenols’. Flavonoids or isoflavonoids are the representatives of the plant polyphenol, and their physiological activities against the human body vary along with the position or number of the hydroxyl groups on their aromatic backbone. For example, one of the major isoflavones in soybean, namely daidzein (7,4′-dihydroxyisoflavone), has moderate antioxidative activity; Also, *ortho*-dihydroxyisoflavone (ODI) derivatives such as 7,3′,4′-trihydroxyisoflavone (3′-hydroxydaidzein), 6,7,4′-trihydroxyisoflavone (6-hydroxydaidzein) and 7,8,4′-trihydroxyisoflavone (8-hydroxydaidzein) display superior antioxidative characters and unique biological functions caused by their actions on different signaling pathways such as anti-skin cancer (3′-hydroxydaidzein), anti-colon cancer, anti-adipogenesis (6-hydroxydaidzein), and anti-atopic dermatitis (8-hydroxydaidzein) pathways (Park et al., 2008; Park et al., 2010; Lee et al., 2011a; Lee et al., 2011b; Seo et al., 2013; Kim et al., 2014). In order to synthesize such hydroxylated polyphenols efficiently, scientists isolated several microbial enzymes and constructed the respective biocatalytic reaction systems. Those studies harnessed microbial cytochrome P450s, flavin-dependent monooxygenases (FMO), or tyrosinases in their natural or mutated forms for an efficient and regioselective production of plant polyphenols (Pandey et al., 2010; Lee et al., 2012; Lee et al., 2014; Lee S.-H. et al., 2016; Lee et al., 2019). In recent studies, a two-component FMO called HpaBC derived from *E. coli* or *P. aeruginosa* has been introduced as another potent candidate to hydroxylate aromatic chemicals. The two component FMO exhibited broad substrate specificities toward plant polyphenols so that naringenin and resveratrol were readily functionalized into eriodictyol (3′-hydroxynaringenin) and piceatannol (3-hydroxyresveratrol), respectively (Furuya and Kino, 2014; Lin and Yan, 2014).

**FIGURE 1:**
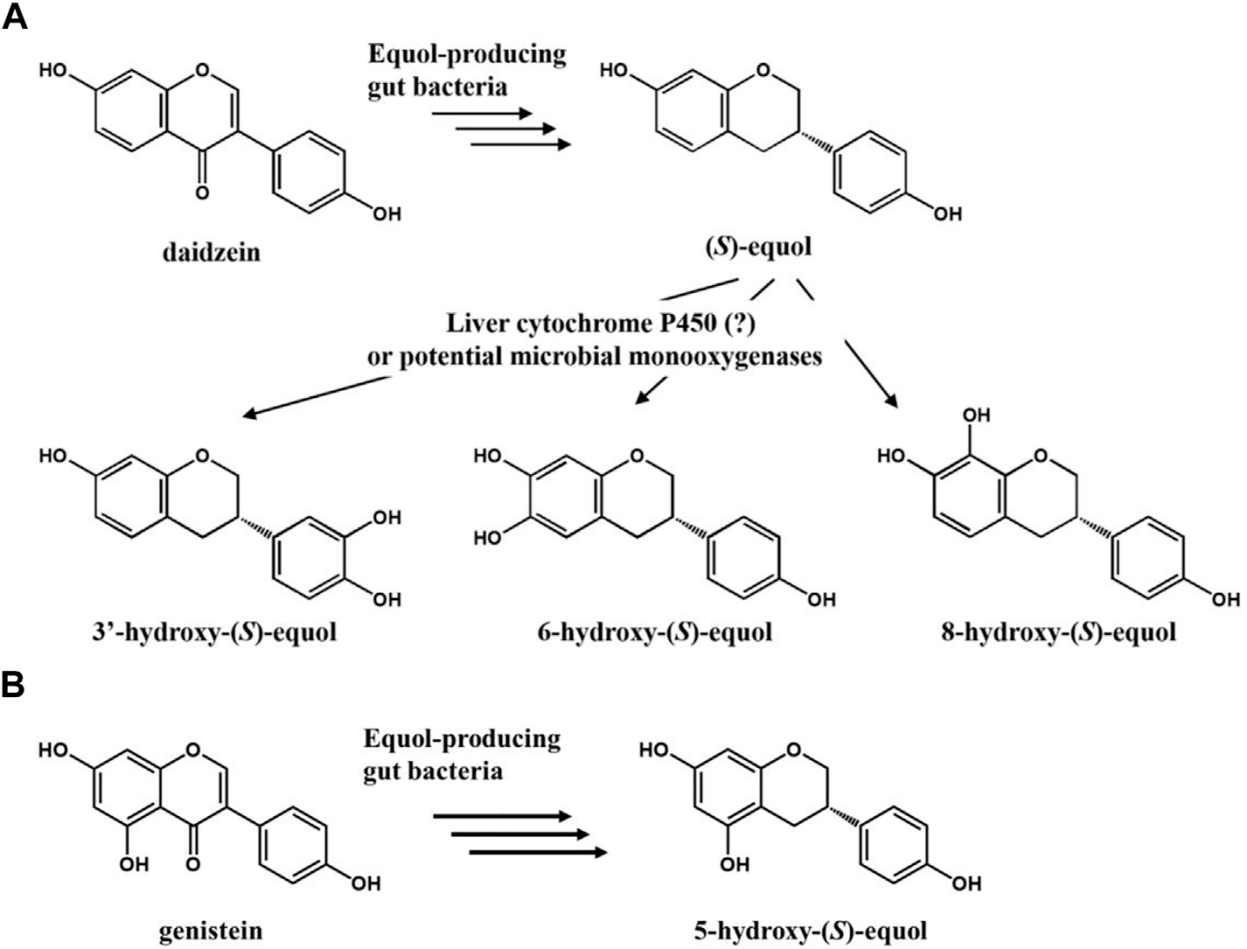
**(A)** Potential scheme of (*S*)-equol hydroxylation after metabolism of daidzein into (*S*)-equol by gut bacteria. **(B)** Metabolism of genistein into 5-hydroxy-(*S*)-equol by gut bacteria.

Herein, we focused on the potential estrogenic and antioxidative activity of HEs which had not been studied due to the lack of proper synthetic methods. One study reported a single-step hydroxylation reaction of equol to 3′HE and 6HE, but the percent yields were quite low (<50%) probably due to the over-oxidation arising from adding too many HpaBC-expressing cells (Hashimoto et al., 2019). To overcome such limitations, herein, a set of polyphenol-hydroxylating enzymes from microbial monooxygenases including cytochrome P450, FMO and HpaBC was tested (Figure 1). Among the candidates, *Ec*HpaBC (HpaBC from *Escherichia coli*) was selected for its relatively high activity toward (*S*)-equol. Then, site-directed mutagenesis was performed for the key residues identified by ligand-docking analysis to modulate its regioselectivity. Finally, regioselective synthesis of 6-hydroxy-(*S*)-equol from daidzein was achieved using recombinant equol-producing and equol-hydroxylating strains simultaneously as whole-cell catalysts.

Other hydroxyequols including 3′-hydroxy-(*S*)-equol (3′HE) and 5-hydroxy-(*S*)-equol (5HE) were also prepared using the same recombinant equol-producing strain (Lee P.-G. et al., 2016; Lee S.-H. et al., 2016; Lee et al., 2017). The synthesized hydroxyequols as well as (*S*)-equol were subjected to the evaluation of their estrogenic activities using yeast two hybrid (Y2H) assay, which examined dose-responsive agonism of two ERs according to interactions with steroid receptor coactivator 1 (SRC1), and DPPH radical scavenging assay was used for the antioxidative efficacy test.

## Results and Discussion

### Screening of (*S*)-Equol-Hydroxylating Monooxygenases

**FIGURE 2:**
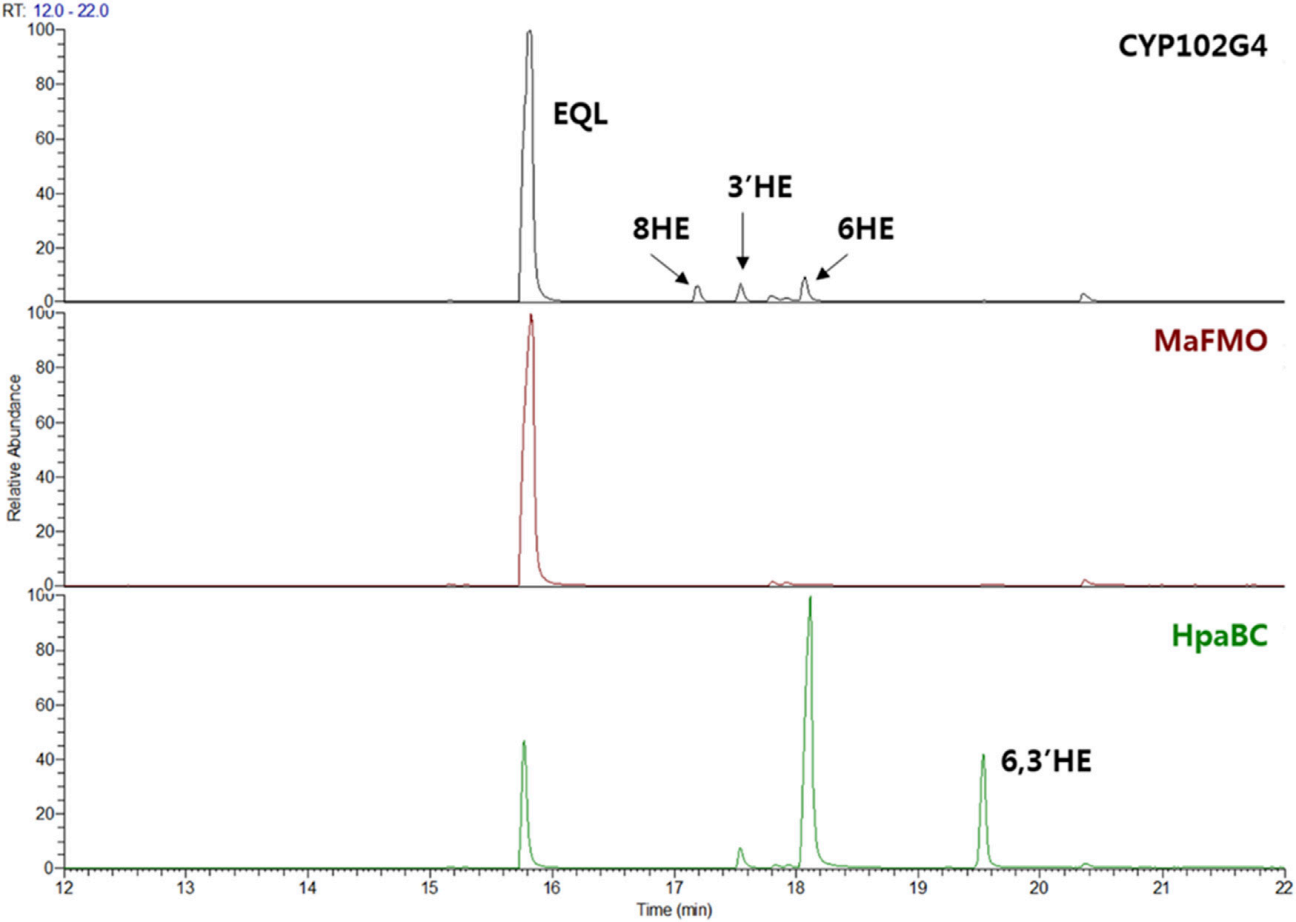
(*S*)-equol (EQL) monooxygenation assay using whole-cells expressing three microbial monooxygenases: CYP102G4, *Ma*FMO, and *Ec*HpaBC. 200 μM of (*S*)-equol was initially used for the whole-cell (OD_600_ = 10) biotransformation, and reaction extracts at 4 h were analyzed using GC-MS.

**FIGURE 3:**
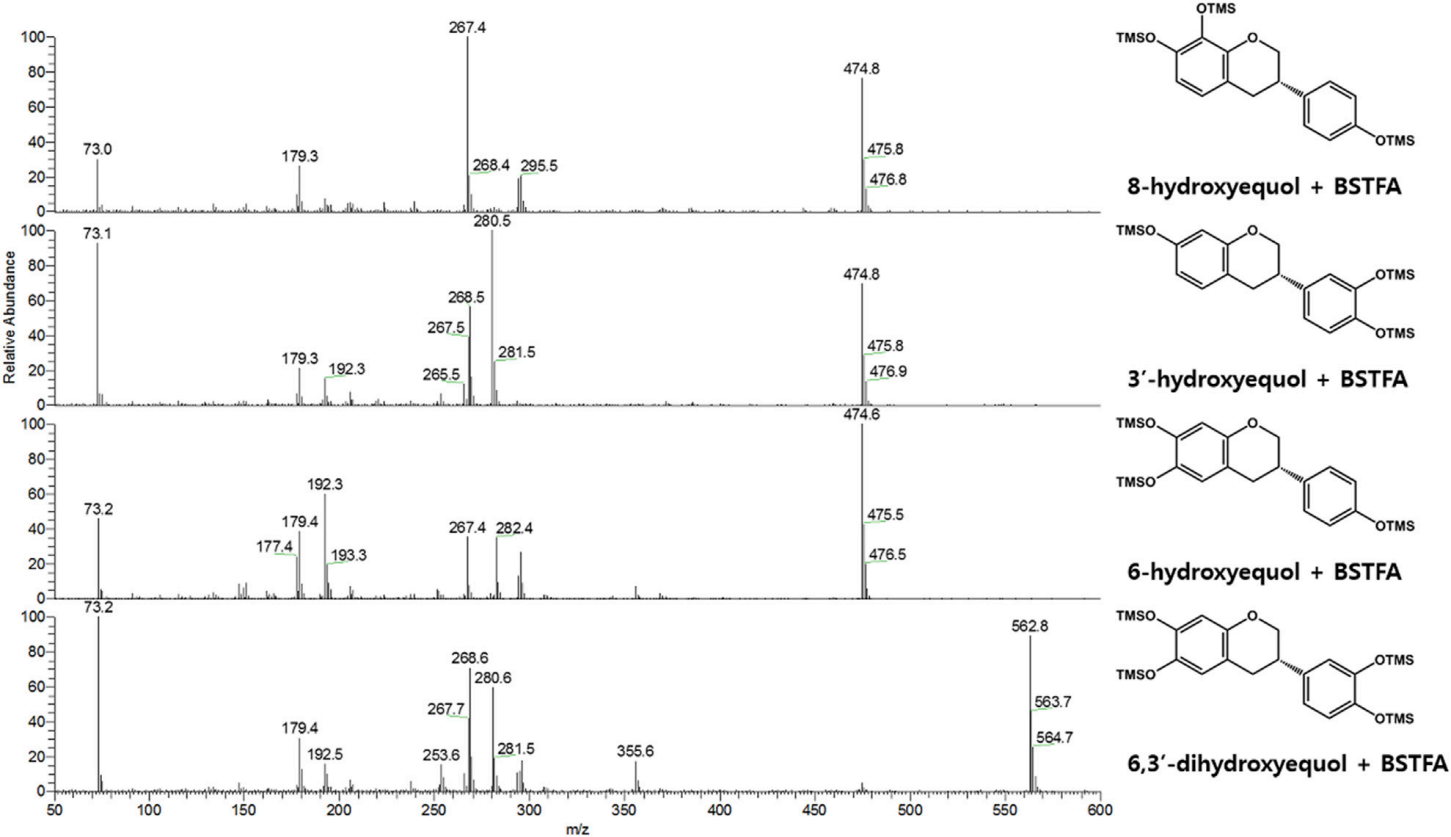
EI-mass spectra (BSTFA: N, O-bis(trimethylsilyl)trifluoroacetamide) of trimethylsilylated hydroxy-(*S*)-equols biosynthesized by CYP0102G4 and *Ec*HpaBC. 3′-hydroxyequol and 6-hydroxyequol were detected in both enzyme reaction samples, while 8-hydroxyequol and 6,3′-dihydroxyequol were detected in the reaction extracts of CYP102G4 and *Ec*HpaBC, respectively.

Since no specific oxygenases responsible for hydroxylation of equol have been reported yet, we examined several microbial monooxygenase candidates including cytochrome P450, FMO, and HpaBC. Because of the unfavorable diphenolase activity, tyrosinase was excluded in this study (Lee et al., 2012). CYP102G4 is a self-sufficient microbial cytochrome P450 isolated from *Streptomyces cattelya*. Similar to other self-sufficient P450s, CYP102G4 directly consumes NAD(P)H as an electron source to activate heme, forming ‘Compound I’ Fe=O (IV) that finally breaks the substrate C-H bond for monooxygenation (Rittle and Green, 2010; Kim et al., 2018). In our previous study, the CYP102G4, was characterized to show a naturally high monooxygenation activity for various polyaromatic substrates including benzophenone and flavone owing to its wide cavity in the active site that enables bulky substrates to easily access the activated heme species (Kim et al., 2018). Whole-cells expressing CYP102G4, however, gave poor conversion of (*S*)-equol (ca. < 1%) with minor detection of 3′-hydroxy-(*S*)-equol (3′HE), 6-hydroxy-(*S*)-equol (6HE), and 8-hydroxy-(*S*)-equol (8HE) as reaction products (Figure 2 and Figure 3).

Next, flavin-containing monooxygenases (FMOs) are potent catalysts to prepare hydroxylated phytochemicals. In former studies, monooxygenation of daidzein, naringenin, and stilibene was achieved using FMOs from various microbial sources (Lee et al., 2014). We selected an FMO derived from *Methylophaga aminisulfidivorans* (*Ma*FMO), which was mainly examined for hydroxylation of indole to form indigo, an aromatic natural dye (Han et al., 2008). Although *Ma*FMO has been shown to exhibit superior activity for indole, no detectable monooxygenation activity for (*S*)-equol was found (Figure 2). Instead, we paid attention to a two-component FAD-dependent monooxygenase called *Ec*HpaBC that was previously identified as a 4-hydroxyphenylacetate 3-monooxygenase in *E. coli* (Prieto and Garcia, 1994). The two-component FMO showed excellent hydroxylation activity for plant-derived polyphenols such as naringenin, resveratrol, and afzelechin with high efficiency (Lin and Yan, 2014; Jones et al., 2016). Interestingly, *Ec*HpaBC-expressing whole cells resulted in remarkable consumption of (*S*)-equol (for OD_600_ = 10, ca. 79% conversion in 4 h) and generated three products with single or multiple hydroxyl group(s), which were finally identified as 6HE, 3′HE, and 6,3′-dihydroxy-(*S*)-equol (6,3′diHE) based on GC-MS analysis (Figure 2 and Figure 3). The EI-MS fragmentation patterns of the hydroxyequols (HEs) corresponded to those of previously identified products, and the fragment ions of *m/z 280* and *295* generated from *m/z 562* mother peak in the 6,3′diHE mass spectrum indicated the presence of hydroxyl groups at C3’ (B-ring) and C6 (A-ring), respectively (Rufer et al., 2006).

The unexpected high conversion rate of *Ec*HpaBC from enterobacterium, *E. coli*, for (*S*)-equol led us to assume that the enzyme might be potentially involved in isoflavone metabolism in the human intestine as well as biodegradation of aromatic compounds by *E. coli* (Diaz et al., 2001). However, a BLAST search of *Ec*HpaB for the human microbiome database (MetaQuery) revealed that the abundance and prevalence of HpaB in the human gut microbiome was only 0.089 and 33.19%, respectively, whereas those of daidzein reductase from *Slackia isoflavoniconvertens* were 0.26 and 100%, respectively. Even though several homologous HpaB proteins were observed in Proteobacteria including genus *Escherichia*, *Klebsiella*, *Enterobacter*, and *Providencia* with >50% sequence identity, the apparent low occurrence of HpaB might lead to infrequent detection of such HEs in the gut. Otherwise, generation of HEs might occur in the human liver *via* human cytochrome P450(CYP) enzymes, and the produced HEs would be estrogenic ligands such as (*S*)-equol or biologically active compounds with different roles from the parent compound (Rufer et al., 2006).

### Modulation of Regioselectivity of HpaB

**FIGURE 4:**
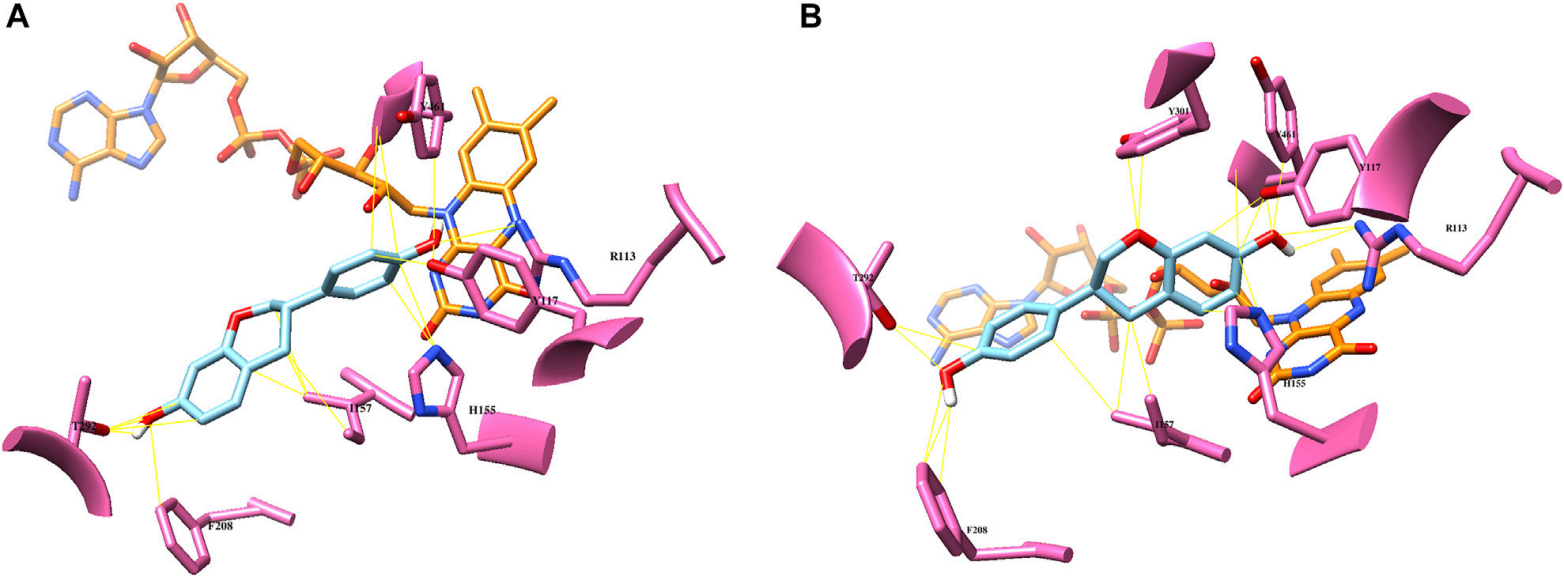
Computational docking analysis of (*S*)-equol (cyan) of the crystal structure of *Ec*HpaB (PDB code: 6QYI) **(A)**: binding mode of 3′-hydroxylation **(B)**: binding mode of 6-hydroxylation. Potential substrate binding residues are highlighted with pink, and the FAD cofactor is shown as orange.

According to the *Ec*HpaBC-WT whole-cell reaction profile, *Ec*HpaBC catalyzed (*S*)-equol into 6HE and 3′HE as major and minor products, respectively, and then, the mono-hydroxylated HEs were subjected to consecutive second hydroxylation, being converted into 6,3′diHE. To interpret the regioselectivity of *Ec*HpaB, biochemical regiospecific analysis of the reaction products was carried out using protein–ligand docking analysis. The two-component HpaBC consists of HpaB, a catalytic component containing flavin adenine dinucleotide (FAD) as a prosthetic group, and HpaC, a NAD(P)H:flavin oxidoreductase component that regenerates FADH_2_ for complete oxidation of the substrate and reduction of molecular oxygen by HpaB (Kim et al., 2007). In a recent report, the well-known broad substrate specificity of *Ec*HpaB toward various aromatic phytochemicals was explained by identifying the crystal structure (PDB code: 6QYI) (Deng et al., 2020). As proposed earlier, FAD(H_2_) freely associates and dissociates in between HpaB and HpaC to complete a cycle of the catalysis, explaining a loosely bound FAD in the active site of *Ec*HpaB (Kim et al., 2007; Deng et al., 2020). Arg164 and Arg333 stabilize diphosphate and adenine moieties, respectively, by constructing a substrate binding site in a groove of *Ec*HpaB. The possible substrate binding site on the *re* face (over flavin) of FAD, partially exposed to the protein surface, was predicted from the binding mode of 4-hydrophenylacetate in *Thermus thermophilus* HB8 HpaB (PDB code: 2YYI) (Kim et al., 2007). Based on the hypothesis that (*S*)-equol would bind to the same *re* face of FAD, energetically minimized (*S*)-equol was docked into the substrate binding region, which resulted in highly probable two binding modes of (*S*)-equol for 3′ and 6-hydroxylation with binding energy -7.6 and -7.4 kcal/mol, respectively (Figure 4). This computational prediction intuitively explains the production of both 3′HE and 6HE by *Ec*HpaBC. Moreover, the generation of another major product of *Ec*HpaBC, 6,3′diHE, could be rationalized by assuming a potential binding of 6HE in the binding pocket for the second hydroxylation at C-3′ and *vice versa* with low probability (Supplementary Figure S1). In the predicted bindings, virtual interactions between the bound substrate and enzyme residues were observed at R113, Y117, H155, I157, T292, and Y301. Among them, R113 and Y117 are the key residues to possibly stabilize 4′- or 7-OH of (*S*)-equol by hydrogen bondings in the respective two binding modes. The other residues might be involved in hydrophobic interactions with the aromatic substrate backbone.

### Site-Directed Mutagenesis of HpaB to Enhance Its Regioselectivity

Given the possible binding mode of (*S*)-equol to *Ec*HpaB, we investigated the role of several active site residues that might determine the regioselectivity of *Ec*HpaB toward (*S*)-equol. Because the native regioselectivity of *Ec*HpaB favored 6-hydroxylation of (*S*)-equol, to generate 6HE as a sole product, we first focused on the effect of the residues contributing to generate 3′HE or 6,3′diHE as minor or major products, respectively. If the residues stabilize the binding mode of (*S*)-equol for 3′-hydroxylation, any possible mutations on the residues might reduce the hydroxylation activity at C3′ at (*S*)-equol (*via* losing hydrogen bonding), while maintaining hydroxylation activity at C6. Because we did not want to lose the robust (*S*)-equol hydroxylation activity of *Ec*HpaB, the residues allowing hydrogen bonds or hydrophobic interactions with (*S*)-equol in both binding modes were excluded from the mutation candidates. An interaction between O1 of 6HE and Y301 was the sole difference between the two binding modes. However, mutation of Y301 might deteriorate the activity of *Ec*HpaB on 6HE because Y301 seems to stabilize the binding of 6HE *via* hydrogen bonding.

**FIGURE 5:**
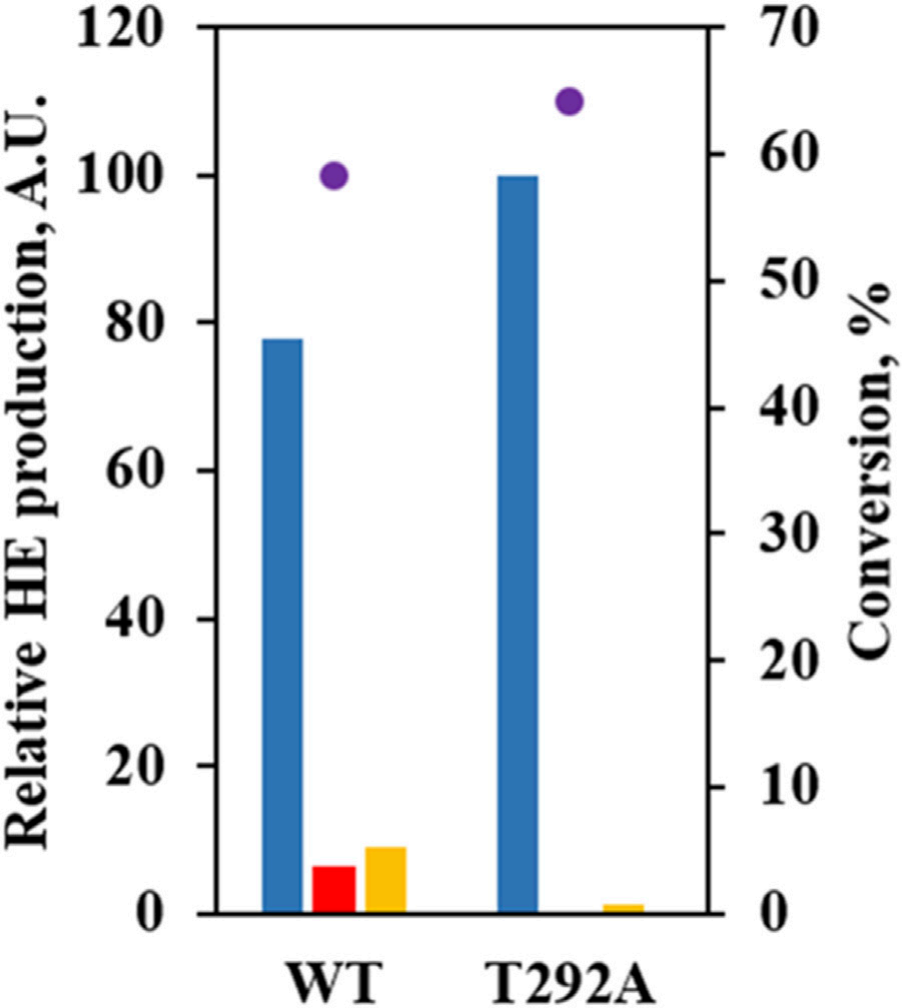
Mutational study to investigate the role of T292 in (*S*)-equol hydroxylations. Relative abundances of 6HE (blue bar), 3′HE (red bar), and 6,3′diHE (yellow bar) were shown with (*S*)-equol conversion at 4 h (purple dot) for *Ec*HpaBC-WT and T292A. The 6HE production level was arbitrarily set to 100, and relative concentrations of the other HEs were estimated with peak areas on gas chromatogram.

Instead, we paid attention to T292 potentially forming a hydrogen bond with 6-OH when 6HE was docked. T292 appeared to contribute more stability to the binding of 6HE than (*S*)-equol due to hydroxylation at C3’ (Supplementary Figure S1). In order to confirm our predictions, the T292A mutant was constructed and evaluated by using whole-cell assay. The mutant showed no detectable production of 3′HE compared to WT, but yielded 6HE as the major product (ca. 98%) and significantly diminished the production of 6,3′diHE (Figure 5), suggesting that the binding of (*S*)-equol or 6HE to wild-type *Ec*HpaB for hydroxylation at C3′ is likely to be stabilized by possible interactions with T292. Especially, hydrogen bonds between 6-OH of 6HE and T292-OH could explain the diminished production of 6,3′diHE by *Ec*HpaB T292A.

### One-Pot Synthesis of *Ortho*-hydroxylated (*S*)-Equols From Daidzein

**FIGURE 6:**
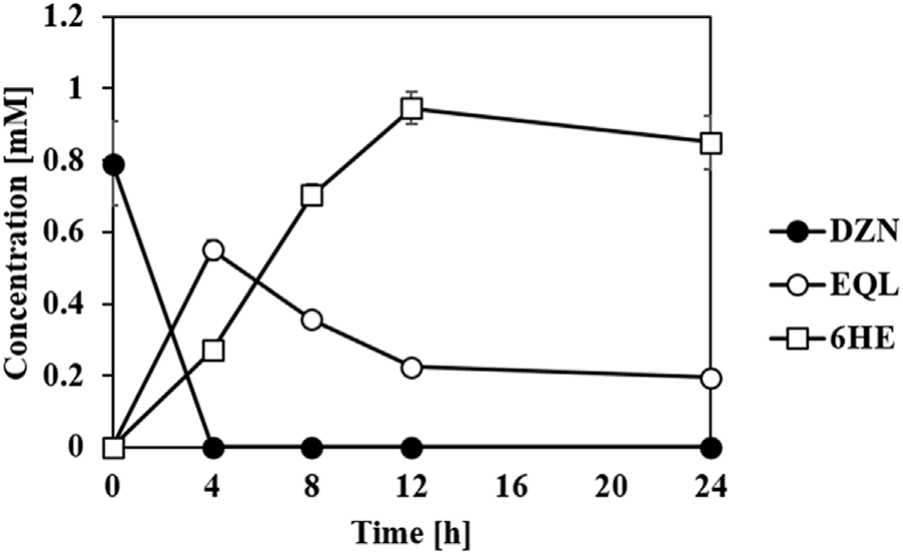
Whole-cell biotransformation of daidzein (DZN) into (*S*)-equol (EQL) and subsequently to 6HE. The tDDDT strain (previously developed by Lee *et al.* (2016)) and t*Ec*HpaBC-T292A strain set up in one-pot reaction catalyzed daidzein (initial concentration: 1 mM) into equol (reduction step), and then subsequently into 6HE (oxidation step) in a highly regioselective manner. All the data were recorded using mean values with standard deviations for triplicates.

In previous studies, we suggested that an equol-producing recombinant *E. coli* strain that converted daidzein into (*S*)-equol would be a useful biocatalytic platform to prepare various equol analogs because the whole-cell biotransformation system of the transformant, tDDDT tolerated aerobic production of equol from daidzein (or 5-hydroxyequol from genistein) and displayed a high yield (g/L titer) and productivity (Lee P.-G. et al., 2016; Lee et al., 2017; Lee et al., 2018). It would be fascinating to synthesize novel HEs rarely found in the human body, but with unknown new biological roles, from common soy isoflavone daidzein. The previous development of the whole-cell catalysts and plasmids drove us to investigate the possibility of a combined use of equol and HE producing strains developed in this study in one-pot reaction. Then, one *E. coli* strain could perform the series of reduction steps of daidzein to (*S*)-equol, and the other strain could subsequently oxidize (*S*)-equol into HEs. The concept has proven to be valid in combinatorial whole-cell biotransformation exploiting tDDDT and the transformant with t*Ec*HpaBC-T292A strains as biocatalysts (Figure 6). All the initial amount of daidzein (ca. 1 mM) was consumed in 4 h, converted into (*S*)-equol rapidly, and then finally 0.95 mM of 6HE (ca. 244 mg/L) was synthesized in a highly regioselective manner. During the whole-cell biotransformation, no significant intermediates or byproducts were detected. This interesting finding shows that *Ec*HpaBC monooxygenase selectively reacts with (*S*)-equol rather than other isoflavone intermediates including daidzein, dihydrodaidzein, and tetrahydrodaidzein, which helped in maintaining the regioselectivity of *Ec*HpaBC-T292A in the microbial cells. This is the first study of one-pot synthesis involving oxidation and reduction of isoflavones using two individual whole cells. Because isoflavone derivatives including equol freely pass through the cell membrane, the two whole-cell compartments can perform simultaneous oxidoreductions of isoflavone with high efficiency (Lee et al., 2017). As a result, it becomes an efficient biocatalytic platform to prepare diverse equol derivatives including hydroxyequols or other chemically modified equols if active equol-catalyzing enzymes are provided. In addition, this platform is an economical choice for producing invaluable hydroxyequol derivatives, for example, the price of non-derivatized (*S*)-equol (>97%, in Sigma, St. Louis, MO, US at 01/10/2022) is $329/25 mg which is about 4.5 fold higher than daidzein (>98%, in Sigma at 01/10/2022, $73.5/25 mg).

### Estrogenic Efficacies of (*S*)-Equol and Hydroxyequol Derivatives

**FIGURE 7:**
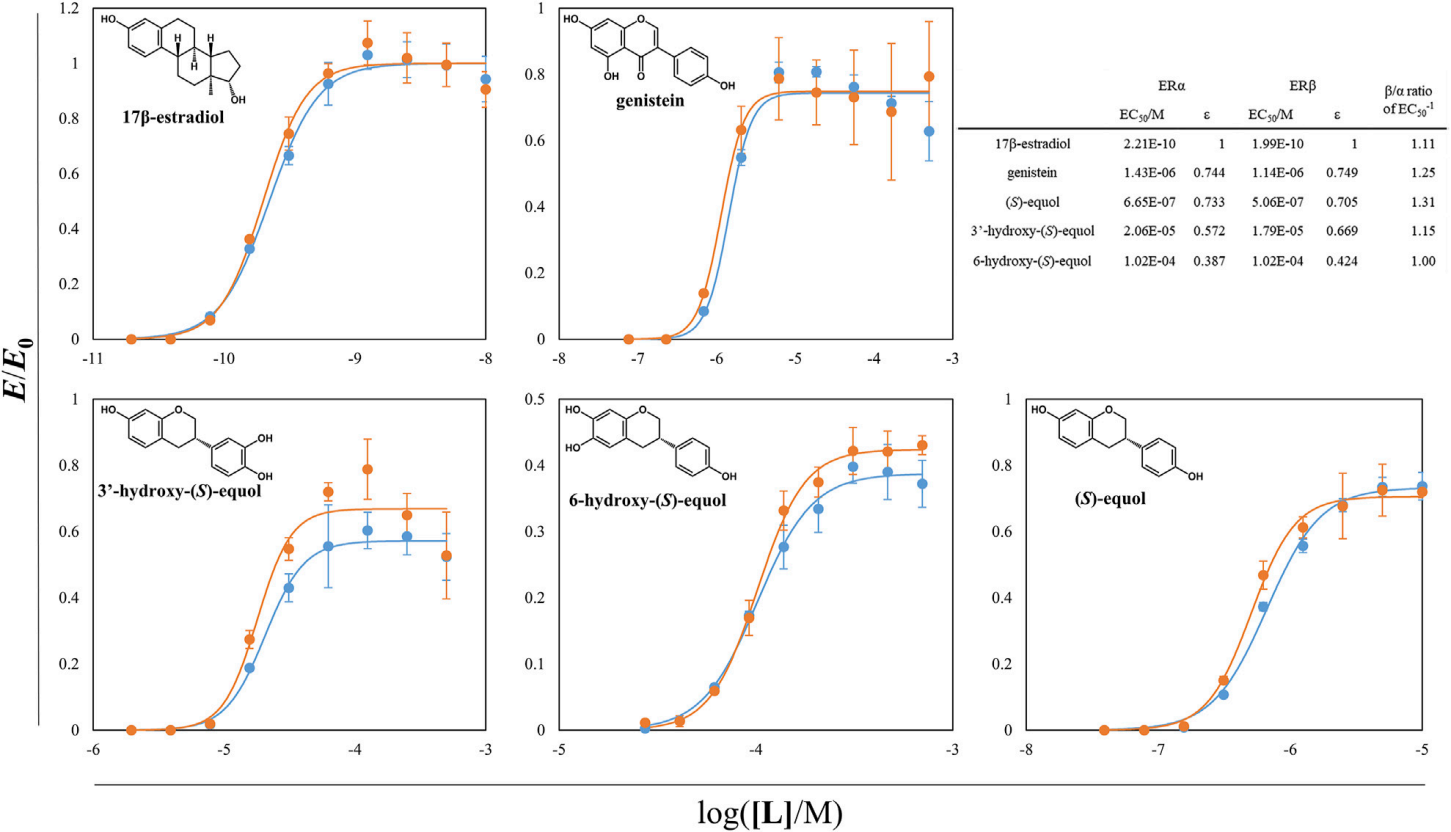
ER-SRC1 agonistic Y2H assay of equol derivatives and the other estrogenic compounds. *ß*-galactosidase activity of yeast L40 cells harboring ER_α_ (or ER_β_) LBD hybrid and tSRC1 hybrid protein was assessed. The normalized responses of ER_α_-SRC1 and ER_β_-SRC1 interaction were expressed as blue and orange circles, respectively. The lines with two different colors were the regressed values. The inward table is the summary of EC_50_s, *ε*s, and *β/α* ratios of each compounds. All the data were recorded using mean values with standard deviations for triplicates.

With the aid of tDDDT whole-cell biotransformation, the other HEs including 3′HE and 5HE were synthesized through the conversion of corresponding isoflavones and purified to evaluate their estrogenic efficacies. The yeast-two-hybrid (Y2H) systems between ERs and SRC1 were exploited and agonistic activities of the estrogenic compounds synthesized throughout this study were measured (Supplementary Figure S3 and Figure 7). In these systems, EC_50_s of 17*β*-estradiol (E2) were about 0.2 nM for two ER isotypes with a slight potency preference on ER_β_. Genistein and (*S*)-equol had about 3-orders of magnitude weaker estrogenic activity both on ER_α_ and ER_β_ than E2 but had SERM activity with the preference to ER_β_ with the β/α ratio of 1.25 and 1.31, respectively. 3′ and 6HE showed much lower activities than the other examined compounds having their EC_50_s with concentrations in the range of 10^–5^∼10^–4^ M and had weaker SERM efficacies than (*S*)-equol displaying *β/α* ratios of 1.15 and 1.00, respectively, indicating that 6HE is a slightly ER_α_ preferring SERM compared to E2. 5HE did not show any response with concentrations in the range of 10^–8^∼10^–3^ M. However, a previous report demonstrated that 5HE had a higher binding affinity to ER_α_ than ER_β_, suggesting that 5HE is an ER_α_-selective antagonist (Lee et al., 2017). In general, all tested HEs did not show prominent estrogenic activity, which suggests that equol loses its estrogenic activity through metabolism in the liver (Rufer et al., 2006).

### Antioxidative Effect of Hydroxyequol Derivatives

**FIGURE 8:**
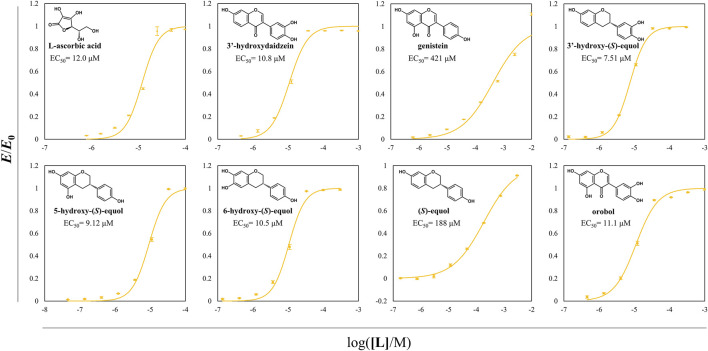
DPPH radical scavenging assay of probable direct antioxidative compounds including HEs. DPPH radical scavenging activity of direct antioxidants was assessed according to the changes in the value of OD_520_/OD_495_. EC_50_ of DPPH radical scavenging of each compound was written in the plots. The normalized OD ratio change values were expressed as yellow circles, and the lines of yellow color were the regressed values. All the data were recorded using mean values with standard deviations for triplicates.

Antioxidative effects of the HEs and the prominent antioxidants were assessed through DPPH radical scavenging assay (Figure 8). Among the examined compounds, the compounds with a catechol moiety showed better radical scavenging activity even among the equol derivatives. Especially, all the HEs of interest had EC_50_ values in the range of 7–11 μM and had superior scavenging activities than 3′HD which is a well-known antioxidative isoflavone. 5HE and 3′HE had lower EC_50_ values than their original isoflavones, suggesting that the backbone of isoflavanes tends to be more potent direct antioxidants than the backbone of isoflavones.

## Conclusion

Despite the possible presence of HEs in the human body, the lack of the synthetic methods of HE has prohibited us from pursuing further studies on their biological roles in hormonal regulation or intended nutritional effect in the human gut microbiome. In this study, we demonstrated that *Ec*HpaBC exhibited significant catalytic activity toward (*S*)-equol (a dominant form of equol enantiomer in the gut), generating 6HE and 6,3′diHE as major products. To enhance the yield of regioselective synthesis of 6HE from (*S*)-equol or daidzein, protein engineering of *Ec*HpaBC using the semi-rational approach, such as ligand-docking analysis and site-directed mutagenesis on the selected target residues, was carried out. Based on this approach, we achieved a 95% yield of 6-hydroxy-(*S*)-equol in 4 h from 1 mM daidzein with a one-pot synthesis procedure. Along with 6HE, the other HEs such as 3′HE and 5HE were synthesized from daidzein and genistein and were assessed for ER binding efficacy and antioxidant efficiency. This work provides valuable information on the construction of an efficient biocatalytic platform to prepare diverse HEs and evaluation of the synthesized HE compounds for ER-binding assays, for the first time. On the basis of our findings, other studies such as the evaluation of HEs’ antagonism and/or *in vivo* testing for exploring novel activities of HEs could be supported, and a facile one-pot high-titer production of another equol derivatives is anticipated.

### Experimental Methods

Chemicals. Daidzein and genistein were purchased from Sigma Aldrich, and (*S*)-equol was purchased from Tocris Bioscience. l-Ascorbic acid was purchased from Junsei. 2,2-Diphenyl-1-(2,4,6-trinitrophenyl)hydrazin-1-yl (DPPH) radical and 17β-estradiol (E2) were purchased from Sigma Aldrich. 3′-Hydroxydaidzein and orobol were prepared from daidzein and genistein respectively using the reported method (Lee S.-H. et al., 2016).

Microbial strains. Cloning information and used primers for the construction of *Ec*HpaBC and *Ma*FMO encoded vectors are listed in Supplementary Table S1. In brief, a two-component flavin-dependent monooxygenase, *Ec*HpaBC, was newly cloned from the genomic DNA of *Escherichia coli* ATCC 8739, and *Ma*FMO was cloned from the genomic DNA of *Methylophaga aminisulfidivorans*. The vector encoding CYP102G4 was previously constructed (Kim et al., 2018). Respective *E. coli* BL21 (DE3) transformants were verified for the soluble expression in SDS-PAGE (Supplementary Figure S2) and were then used as microbial strains for the whole-cell biotransformation. Three *Ec*HpaB mutants were made by site-directed mutagenesis using the listed overlapping primer-containing intended mutations. *Saccharomyces cerevisiae* L40 (*MATa his3*-200 *trp1*-901 *leu*2-3,112 *ade2 lys2*-801a.m. *URA3*(*lexAop*)8- *lacZ LYS2*(*lexAop*)4-*HIS3*) strain was utilized as a host for yeast-two-hybrid (Y2H) assays. The bait hybrid proteins were cloned in the pBTM116 vector as fusions of LexA DNA-binding domain (DBD)-human estrogen receptor *a* ligand-binding domain (ER_α_ LBD, 312–595) or LexA DBD-ER_β_ LBD (264–510). The prey hybrid protein was cloned in the pVP16 vector as a fusion of the VP16 activator domain (AD)-(Gly_4_Ser)_2_ linker-truncated steroid receptor coactivator 1 (tSRC1) (613–773). These sets of two vectors (pBTM116:LexA DBD-ER_α_ LBD & pVP16:VP16 AD-(Gly_4_Ser)_2_-tSRC1 or pBTM116:LexA DBD-ER_β_ LBD & pVP16:VP16 AD-(Gly_4_Ser)_2_-tSRC1) were transformed in the L40 strains.

Whole-cell biotransformation (GC-MS analysis). The aforementioned *E. coli* transformants were grown overnight, 1 ml of which was transferred to 50 ml of LB containing appropriate antibiotics. When the optical density (OD_600_) of the cells reached 0.6 to 0.8, addition of 0.1 mM isopropyl-thio-β-d-galactopyranoside (IPTG) or 0.5 mM 5-aminolevulinic acid for heme formation of CYP102G4 induced the heterologous expression of monooxygenases overnight at 18°C. The monooxygenase-expressing cells were harvested *via* centrifugation (4°C, 4,000 rpm; 3,480 rcf for 10 min), washed with phosphate buffer saline (0.5x volume), and then centrifuged again for subsequent whole-cell catalysis.

The prepared whole cells were resuspended in the reaction solution (final OD_600_ = 10, total volume = 5 ml) composed of 0.1 M potassium phosphate buffer (KPB) pH 7.0, 1% (w/v) glucose, 5 mM l-ascorbic acid, and 0.2 mM (*S*)-equol. Whole-cell biotransformation was performed in the baffled flask shaken by 180 rpm at 30°C. In the case of consecutive oxidoreduction of daidzein into HEs, the reaction condition was slightly modified by referring to apreviously reported study (0.2 M KPB pH 8.0, 2% (w/v) glucose, 1% (v/v) glycerol, and polyvinylpyrrolidone 1% (w/v) in a 140 rpm shaking incubator) (Lee et al., 2018). The equol-producing whole-cells were also prepared following the previously reported method (Lee P.-G. et al., 2016).

Preparation of hydroxyequols. 6-Hydroxyequol (6HE) was synthesized with the aforementioned method. 5HE was synthesized with the previously reported method using genistein as the initial substrate (Lee P.-G. et al., 2016; Lee et al., 2017). 3′HE was synthesized using the same method from 3′-hydroxydaidzein which was prepared by *ortho*-hydroxylation of the tyrosianse derived from *Bacillus megaterium* with borate chelation (Lee P.-G. et al., 2016; Lee S.-H. et al., 2016). All of the isoflavonoids were purified through EA extraction, HPLC preparation (Econosil C18 prep column, 10 μm, 22 × 250 mm; Alltech, United States), and freeze-drying. The final purity of 6HE, 3′HE, and 5HE was confirmed with GC-MS or HPLC and ^1^H-NMR analysis.

6-Hydroxy-(*S*)-equol: ^1^H-NMR [(CD_3_)_2_SO, 400 MHz] δ 2.73 (m, 2H, H-4), 2.98 (m, 1H, H-3), 3.81 (t, *J* = 10.4 Hz, 1H, H-2_β_), 4.05 (m, 1H, H-2_α_), 6.19 (s, 1H, H-8), 6.44 (s, 1H, H-5), 6.71 (d, *J* = 8.4 Hz, 2H, H-3′), 7.09 (d, *J* = 8.4 Hz, 2H, H-2’), 8.26 (s, 1H, OH), 8.69 (s, 1H, OH), and 9.26 (s, 1H, OH).

5-Hydroxy-(*S*)-equol: ^1^H-NMR [(CD_3_)_2_SO, 400 MHz] δ 2.47 (dd, *J* = 16.1, 10.9 Hz, 1H, H-4_β_), 2.73 (ddd, *J* = 16.1, 5.5, 1.4 Hz, 1H, H-4_α_), 2.95 (m, 1H, H-3), 3.83 (t, *J* = 10.4 Hz, 1H, H-2_β_), 4.10 (dq, *J* = 10.4, 1.6 Hz, 1H, H-2_α_), 5.70 (d, *J* = 2.3 Hz, 1H, H-6), 5.90 (d, *J* = 2.3 Hz, 1H, H-8), 6.72 (d, *J* = 8.5 Hz, 2H, H-3′), 7.11 (d, *J* = 8.5 Hz, 2H, H-2’), 8.93 (s, 1H, OH), 9.17 (s, 1H, OH), and 9.25 (s, 1H, OH).

3′-Hydroxy-(*S*)-equol: ^1^H-NMR [(CD_3_)_2_SO, 400 MHz] δ 2.78 (m, 2H, H-4), 2.94 (m, 1H, H-3), 3.87 (t, *J* = 10.5 Hz, 1H, H-2_β_), 4.15 (m, 1H, H-2_α_), 6.19 (d, *J* = 2.4 Hz, 1H, H-8), 6.29 (dd, *J* = 8.2, 2.4 Hz, 1H, H-6), 6.56 (dd, *J* = 8.2, 2.2 Hz, 1H, H-6′), 6.67 (d, *J* = 2.3 Hz, 1H, H-2′), 6.69 (d, *J* = 8.2 Hz, 1H, H-5′), 6.87 (d, *J* = 8.2 Hz, 1H, H-5), 8.78 (s, 2H, OH), and 9.14 (s, 1H, OH).

Ligand-docking simulation. AutoDock Vina 1.1.2 (Trott and Olson, 2010) predicted docking modes of (*S*)-equol or 6HE in the reported *Ec*HpaB crystal structure (PDB code: 6QYI) (Deng et al., 2020), which were visualized by using Chimera 1.15.

Bioinformatic analyses. MetaQuery, an open-source web server for quantitative analysis of a given gene in the human gut microbiome, was utilized to calculate the abundance and prevalence of HpaB (from *E. coli*) and daidzein reductase (from *Slakia isoflavoniconvertens*, reference) in a human gut microbiome database (Nayfach et al., 2015). The minimum percent identity/maximum E-value/query alignment coverage/target alignment coverage were set to 30%/1e-5/70%/70% (web default values), respectively.

Yeast-two-hybrid assay. The transformed L40 strains were grown in synthetic leucine and tryptophan drop-out (SD L^−^W^-^) media. In 96-deep well plates, each well with the cultural medium of 500 μL SD L^−^W^-^ contained initial OD_600_ = 0.1 of L40 preculture, 2% (v/v) DMSO, and specific amount of isoflavonoids or 17β-estradiol (E2). These plates were incubated at 30 °C with shaking of 1,000 rpm for overnight (∼20 h). After the incubation, the cultures were centrifuged, and the supernatants were discarded. In each well, 500 μL Z-buffer (60 mM Na_2_HPO_4_, 40 mM NaH_2_PO_4_, 10 mM KCl, and 1 mM MgSO_4_; pH 7.0) with 1 mM dithiothreitol (DTT) was filled and resuspended. 50 μL of each suspension was taken for OD_600_ measurement before chloroform treatment. (This value was not used for the measurement of the Miller unit.) Chloroform 50 μL was added to each 450 μL of the suspension, and the solution mixture was shaked for 1 min at 1,000 rpm to disrupt the cell membrane. The disrupted suspensions were kept still at room temperature for 2 min, and 50 μL aqueous aliquots were taken. In polystyrene flat-bottomed 96-well plate, each well was charged with 100 μL of Z-buffer with 1 mM DTT, 50 μL aqueous aliquot, and 50 μL of Z-buffer with 1 mM DTT and 0.4 mg/ml *ortho*-nitrophenyl-β-galactoside (ONPG). Initially, OD_600_ of all the wells was measured, and subsequently, OD_420_ of the same wells was measured for 30 min with a minute interval. The Miller units (dOD_420_/d*t*/OD_600_) of all the wells were calculated and normalized by using the value of the positive control (10 nM E2). The data were triplicated, and the points of average values were regressed using the following formula (Buchwald, 2020).

$$\frac{E}{E_{0}}=\frac{\varepsilon }{1+10^{n(\log\left({\text{EC}}_{50}\right)-\text{log}\left(\left[\text{L}\right]\right))}},$$

where *E*/*E*_0_ is the normalized Miller unit and *ε* is the maximum response relative to E2 (*ε*(E2) = 1). [L] is the concentration of ligands and *n* is the Hill’s coefficient. EC_50_ is the value of effective concentration at *E*/*E*_0_ = 0.5*ε*.

DPPH radical scavenging assay. In each well of polystyrene flat-bottom 96-well plates, 200 μL of solution containing 0.1 mM 2,2-diphenyl-1-(2,4,6-trinitrophenyl)hydrazin-1-yl (DPPH) radical, 50% (v/v) MeOH, 10% (v/v) DMSO, specific amount of compounds, and 1x concentration of phosphate buffered saline (PBS) was made. The PBS was prepared with 4x concentration of its original recipe with pH 7.4 and diluted by adding 50 μL in the solution mixture. The plates were incubated at 37°C for 30 min. The OD_520_ and OD_495_ of incubated plates were measured. The radical scavenging activity was defined as (OD_520_/OD_495_)_DMSO_- OD_520_/OD_495_. (OD_520_/OD_495_)_DMSO_ is the OD_520_/OD_495_ of the DMSO control without antioxidative compounds. The radical scavenging activity values were normalized with the value of the positive control (1 mM of l-ascorbic acid). The data were triplicated, and the average values were regressed using the following formula.

$$\frac{E}{E_{0}}=\frac{1}{1+10^{n(\log\left({\text{EC}}_{50}\right)-\text{log}\left(\left[\text{L}\right]\right))}},$$

where *E*/*E*_0_ is the normalized radical scavenging activity, [L] is the concentration of compounds, and *n* is the Hill’s coefficient, and EC_50_ is the value of effective concentration at *E*/*E*_0_ = 0.5.

## Data Availability

The original contributions presented in the study are included in the article/Supplementary Material; further inquiries can be directed to the corresponding author.
