# Supplementary material for: Regioselective One-Pot Synthesis of Hydroxy-(S)-Equols Using Isoflavonoid Reductases and Monooxygenases and Evaluation of the Hydroxyequol Derivatives as Selective Estrogen Receptor Modulators and Antioxidants
Source: Front Bioeng Biotechnol. 2022 Mar 24;10:830712. doi: 10.3389/fbioe.2022.830712 (PMC8987157; doi:10.3389/fbioe.2022.830712)
Supplement: Supplementary file 1 [file DataSheet1.docx]

**Supplementary Materials**

**Regioselective One-pot Synthesis of Hydroxy-(*S*)-equols Using Isoflavonoid Reductases and Monooxygenases, and Evaluation of the Hydroxyequol Derivatives as Selective Estrogen Receptor Modulators and Antioxidants.**

Hanbit Song^1,2†^, Pyung-Gang Lee^1,2,3†^, Junyeob Kim^1,2^, Joonwon Kim^1,2^, Sang-Hyuk Lee^1,2^, Hyun Kim^1,2^, Uk-Jae Lee^1,2^, Jin Young Kim^1,2^, Eun-Jung Kim^4^, Byung-Gee Kim^1,2,4,5^*

^1^School of Chemical and Biological Engineering, Seoul National University, Seoul, 08826, South Korea

^2^Institute of Molecular Biology and Genetics, Seoul National University, Seoul, 08826, South Korea

^3^Institute of Engineering Research, Seoul National University, Seoul, 08826, South Korea

^4^Bio-MAX/N-Bio Institute, Seoul National University, Seoul, 08826, South Korea

^5^Institute for Sustainable Development (ISD), Seoul National University, Seoul, 08826, South Korea

^†^Hanbit Song and Pyung-Gang Lee contributed equally to this work.

*Corresponding author

Tel: +82-2-880-6774; Fax: +82-2-876-8945

E-mail: byungkim@snu.ac.kr (B. G. Kim)


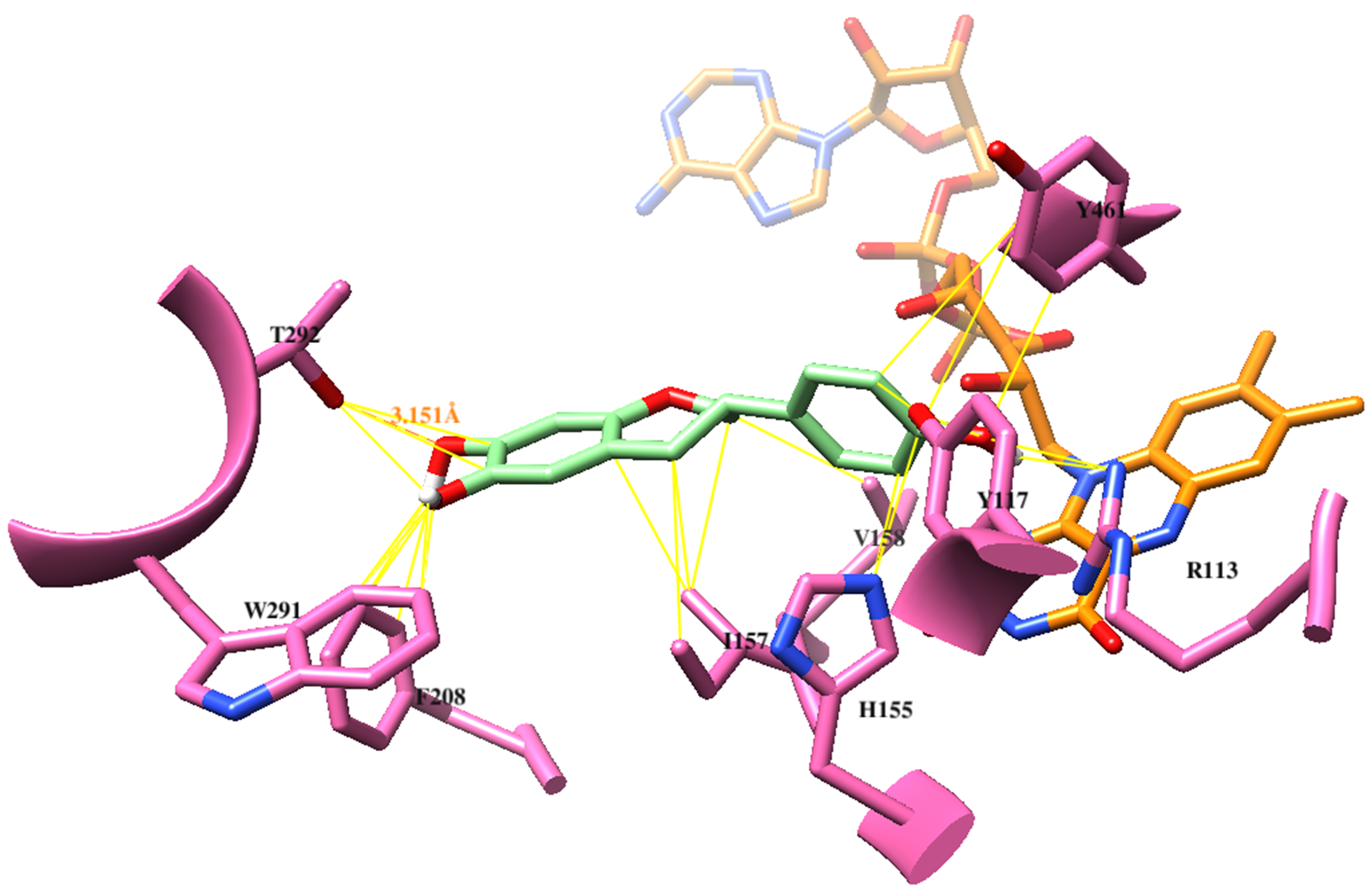


**Figure S1. Docking results of 6HE into *Ec*HpaB.**

A potential hydrogen bond between 6HE and T292 was shown with orange dashed line.

**Table S1. Cloning information and primers for constructing microbial strains.**

| **Cloning information** | | |
| --- | --- | --- |
| **Cloning proteins** | **Restriction enzyme** | **Primer sequences (5’ to 3’)** |
| EcHpaB  (pETDuet-1, MCS-2) | NdeI  (forward) | GGGAAA **CATATG** CATCACCATCATCACCAC AAACCAGAAGATTTCCGCGC |
|  | XhoI  (reverse) | GGGAAA **CTCGAG** TTA TTTCAGCAGCTTATCCAGCATGTTG |
| EcHpaC  (pETDuet-1, MCS-1) | BamHI  (forward) | GGGAAA **GGATCC** G CAATTAGATGAACAACGCCTGC |
|  | NotI  (reverse) | GGGAAA **GCGGCCGC** TTA AATCGCAGCTTCCATTTCCAGC |
| MaFMO  (pET28a) | NheI  (forward) | GGGAAA **GCTAGC** ATGGCAACTCGTATTGCGATAC |
|  | XhoI  (reverse) | GGGAAA **CTCGAG** TTA AGCTTCTTTAGCCACAGG |
| **Transformant notation** | | |
| **Name** | | **Plasmid description** |
| tDDDT | | pRSFDuet-1(*dznr/ddrc*) + pCDFDuet-1(*dhdr/thdr*) |
| tEcHpaBC-WT | | pETDuet-1(*hpaC/hpaB wild-type*) |
| tEcHpaBC-T292A | | pETDuet-1(*hpaC/hpaB T292A*) |
| **Site-directed mutagenesis of EcHpaB** | | |
| **Primer name** | | **Primer sequences (5’ to 3’)** |
| T292A-F | | CGTCGCTGG **GCA** ATGGAAGGCG |
| T292A-R | | CTTCCAT **TGC** CCAGCGACGGC |
| S464A-F | | CTACTCCGGT **GCC** CAGGATGAGATTC |
| S464A-R | | TCATCCTG **GGC** ACCGGAGTAGTTG |
| R469A-F | | GATGAGATT **GCC** CTGCAGTGTC |
| R469A-R | | CACTGCAG **GGC** AATCTCATCCTG |


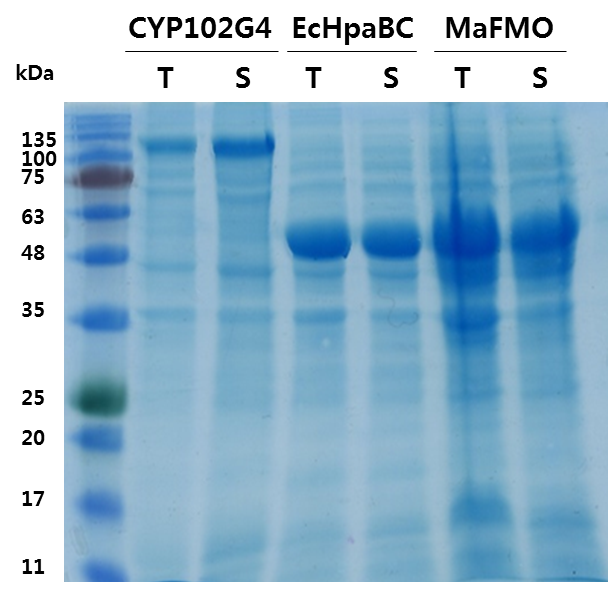


**Figure S2. Heterologous expression of three microbial monooxygenases in *E. coli*.**

Harvested *E. coli* whole-cells heterologously expressing the three monooxygenases were resuspended in 5 ml of 0.1M KPB (pH 7.0), then lysed using sonicator (3 sec on, 8 sec off) in an ice-cooled bottle. The cell lysates (total; T) were centrifuged at 14,000 x g for 30 min at 4ºC, of which supernatants (soluble; S) were also analyzed on SDS-PAGE. The theoretical protein sizes are 117 kDa (CYP102G4), 60 kDa (EcHpaB), 20 kDa (EcHpaC) and 55 kDa (MaFMO).


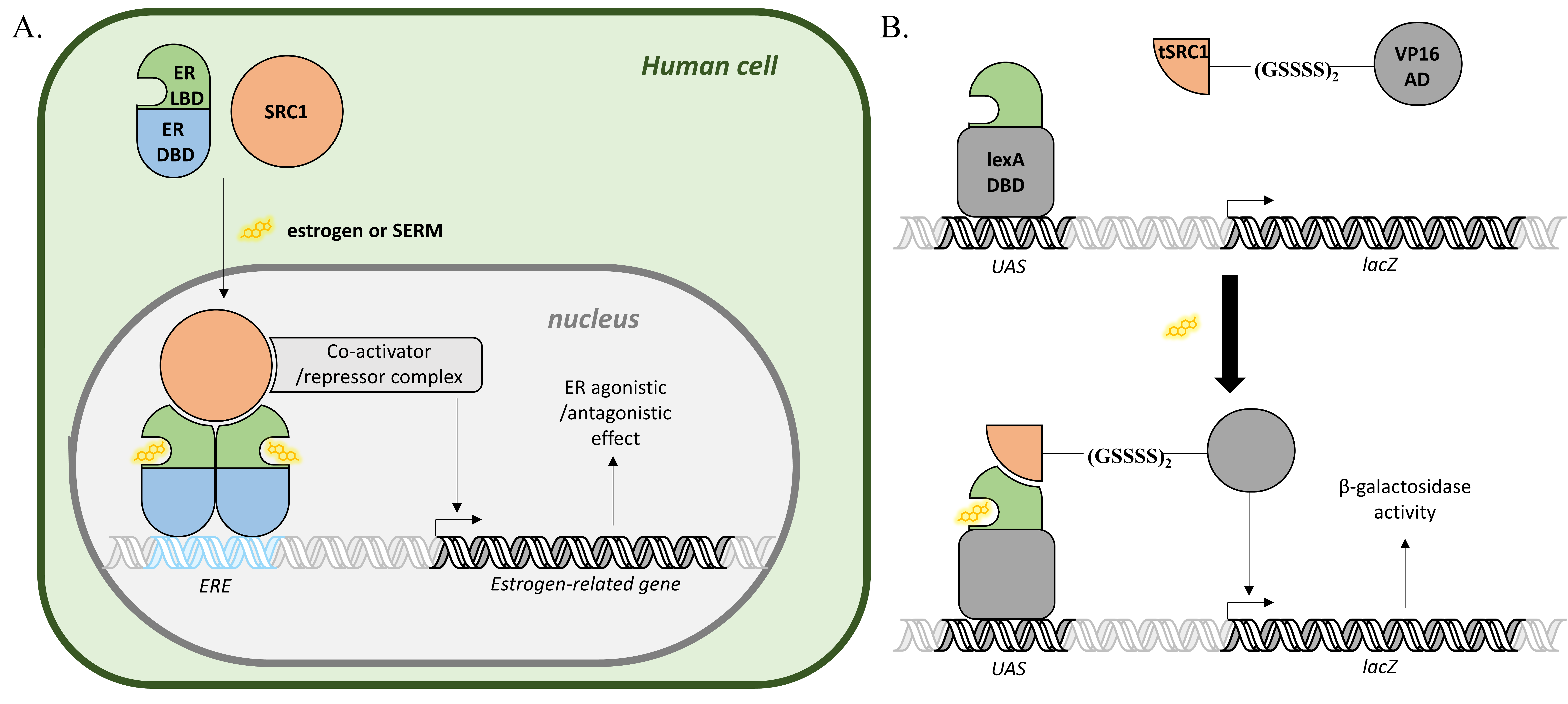


Figure S3. Working principle of ER-SRC1 yeast-two-hybrid assay.

Given agonistic or antagonistic mechanism of ER (α or β)-SRC1 complex in human cells (A), agonistic yeast-two-hybrid assay was constructed exploiting the interaction between ER (α or β) and tSRC1 (B).

.
